# Antibiotic Usage and Resistance in Food Animal Production: What Have We Learned from Bangladesh?

**DOI:** 10.3390/antibiotics10091032

**Published:** 2021-08-24

**Authors:** Sukanta Chowdhury, Sumon Ghosh, Mohammad Abdul Aleem, Shahana Parveen, Md. Ariful Islam, Md. Mahbubur Rashid, Zubair Akhtar, Fahmida Chowdhury

**Affiliations:** 1 International Centre for Diarrhoeal Disease Research, Bangladesh (icddr,b), Dhaka 1212, Bangladesh; sumon.ghosh@icddrb.org (S.G.); drmdaleem@icddrb.org (M.A.A.); shahana@icddrb.org (S.P.); arif@icddrb.org (M.A.I.); mahbubur.rashid@icddrb.org (M.M.R.); zakhtar@icddrb.org (Z.A.); fahmida_chow@icddrb.org (F.C.); 2 School of Population Health, University of New South Wales (UNSW), Sydney, NSW 1466, Australia

**Keywords:** food animals, antibiotic usage, antibiotic resistance, Bangladesh

## Abstract

Irrational and inappropriate use of antibiotics in commercial chicken and aquaculture industries can accelerate the antibiotic resistance process in humans and animals. In Bangladesh, the growing commercial chicken and aquaculture industries are playing significantly important roles in the food value chain. It is necessary to know the antibiotic usage practices and antibiotic resistance in food animal production to design rational policies, guidelines, and interventions. We conducted a narrative review to understand the level of antibiotic usage and resistance in food animal production in Bangladesh. Information about antibiotic usage in different food animal production systems, including commercial chickens and aquaculture in Bangladesh is inadequate. Only a few small-scale studies reported that the majority (up to 100%) of the broiler and layer chicken farms used antibiotics for treating and preventing diseases. However, numerous studies reported antibiotic-resistant bacteria of public health importance in commercial chicken, fish, livestock, and animal origin food. The isolates from different pathogenic bacteria were found resistant against multiple antibiotics, including quinolones, the third or fourth generation of cephalosporins, and polymyxins. Veterinary practitioners empirically treat animals with antibiotics based on presumptive diagnosis due to inadequate microbial diagnostic facilities in Bangladesh. Intensive training is helpful to raise awareness among farmers, feed dealers, and drug sellers on good farming practices, standard biosecurity practices, personal hygiene, and the prudent use of antibiotics. Urgently, the Government of Bangladesh should develop and implement necessary guidelines to mitigate irrational use of antibiotics in food animals using a multi-sectoral One Health approach.

## 1. Introduction

Globally, antimicrobial resistance (AMR) is one of the greatest threats to public health. Irrational and inappropriate usage of antimicrobials in humans, poultry, fish, and livestock are important drivers to AMR emergence. The increasing AMR pattern subsequently leads to treatment failure, causing significant morbidity and mortality and additional healthcare costs annually [1,2]. Antimicrobials are frequently used as prophylactic drugs in the commercial animal production system in low–middle–income countries [3,4,5]. Antimicrobial consumption (AMC) in the animal production system is almost double human consumption [6]. In many countries, antimicrobials are widely available to humans and animals without any restriction. Unqualified animal healthcare providers play an important role in using antimicrobials in food-producing animals in developing countries [7].

The World Organization for Animal Health (OIE) recommended a list of antimicrobials for veterinary use. Antimicrobial agents are classified into three categories; Veterinary Critically Important Antimicrobial Agents (VCIA), Veterinary Highly Important Antimicrobial Agents (VHIA), and Veterinary Important Antimicrobial Agents (VIA). The third and fourth generations of cephalosporins and fluoroquinolones are considered critically important antimicrobials for human and animal health. OIE also recommended avoiding antimicrobial for prophylactic purposes in the absence of clinical signs in the animals [7].

The European Medicines Agency (EMA) classified antibiotics into four categories considering the risk of spreading resistance from animals to humans. Category A includes antibiotics that are not authorized in veterinary medicine but authorized in human medicine. Category B is critically important for human health and includes quinolones (fluoroquinolones and other quinolones), the third or fourth–generation cephalosporins (excluding those with beta-lactamase inhibitors), and polymyxins. Category B should be prescribed when no other alternative antibiotics in Categories C or D are found effective [8]. 

Drivers of antibiotic resistance are complex in low-middle-income countries, and it requires multi-sectoral involvement to control the spread of AMR pathogens in both humans and animals. A trans-disciplinary One Health approach is necessary to detect AMR’s emergence, identify AMR drivers, and develop interventions to mitigate the AMR in humans and animals [9]. Food and Agriculture Organization (FAO) of the United Nations (UN), the World Organization for Animal Health (OIE), and the World Health Organization (WHO), and other professional agencies recognized the multi–sectoral One Health approach to address the public health threat of animal origin [10,11]. In 2015, WHO established the Global Antimicrobial Resistance and Use Surveillance System (GLASS) to facilitate national AMR and antimicrobial consumption (AMC) surveillance systems and data sharing through a collaborative relationship between the human, animal, and environmental sectors. Bangladesh is one of the participating countries to share AMR surveillance data using the One Health approach [12].

In Bangladesh, commercial chicken and aquaculture industries are expanding day by day to meet the increasing demand for animal-source nutrition for humans. Both sectors are playing a significantly important role in the food value chain. The commercial chicken industry mainly includes broiler, layer, and Sonali (a cross-breed of Rhode Island Red cocks and Fayoumi hens) intensive farms, whereas commercial aquaculture comprises ponds (close culture system), tanks, net pens, and cage cultures [13,14]. Around 20% of rural households have ponds in their homestead, and 30% of total fish production comes from commercial extensive, semi-intensive, and intensive farms [15]. Commercial chicken and fish farms perform intensive operations to increase production and minimize disease prevalence. Many types of drugs, including antimicrobials, vitamins, minerals, and antimicrobial growth promoters are extensively used in commercial chicken and aquaculture production sectors [16,17,18,19,20,21]. Despite the massive benefit of treating animal diseases using antimicrobial drugs, the resulting emergence of antibiotic resistance has raised global concerns [22]. Many farmers in Bangladesh are less aware of the negative impact of excessive, irrational, and prophylaxis use of antibiotics in animals, and aquaculture. Inadequate veterinary healthcare facilities, insufficient monitoring and regulatory services on antibiotic usage, high occurrence of diseases, and malpractices by unqualified veterinary healthcare providers (quack, drug sellers, and animal feed dealers) contributed a crucial role in the increased and misusage of antibiotics in animal health sectors [21]. 

To contain AMR, the government of Bangladesh approved National Strategy for AMR Containment (ARC): 2017–2021. The objectives of this strategy are to establish a multi–sectoral One Health approach to plan, coordinate, and implement ARC containment activities, ensure rational use of antimicrobial agents in humans and animals, strengthen infection prevention and control measures, strengthen bio-safety and bio-security practices, strengthen the surveillance system for AMR and promote operational research, strengthen regulatory provisions, and establish advocacy, communication, and social mobilization [23]. The Government of Bangladesh formulated the first National Drug Policy in 1982 to ensure the drug safety, quality, and control of drug prices for human health [24]. However, there is no such drug policy or guideline for the animal production sectors. To stop using antibiotics in animal feed during manufacturing, the Bangladesh government constituted a law named “Bangladesh Fish Feed and Animal Feed Act 2010” [25]. Thus far, no study was carried out to determine the existence of antibiotics in animal feed. A significant portion of the antibiotics used in animal production sectors is procured from outside the country. The Directorate General of Drug Administration (DGDA) under the Ministry of Health and Family Welfare monitors regulates the import, packaging, production, licensing, and registration of antibiotics [26].

In Bangladesh, minimal information is available to describe the antibiotic usage practices in food-producing animals. On the other hand, several studies were carried out to examine antibiotic resistance. Antibiotic resistance, particularly in commercial chicken production, was explored more than other animal production sectors, including aquaculture and livestock. Notably, to date, the country has no separate policy or guideline for antibiotic usage in animal sectors. More updated information on the extent of antibiotic usage and resistance in food-producing animals is crucial to understand the current situation and design proper interventions to minimize the risk of antibiotic resistance in animals and humans. This narrative review provides an overview of antibiotic usage and antibiotic resistance in food-producing animals in Bangladesh. The findings of this review would help policymakers, animal health experts, and animal as well as fish farmers to understand the antibiotic usage and antibiotic resistance-related problems, which are essential to develop effective policies and guidelines for rational antibiotic use to protect animals as well as human health.

## 2. Materials and Methods

We conducted a web-based narrative review of the published literature focusing on antibiotic use and antibiotic resistance in animals reported from Bangladesh. We performed a comprehensive literature review to find relevant research articles, review articles, abstracts, case reports, communications, letters, book chapters, conference proceedings, and other relevant documents through PubMed, Google Scholar, Medline, and PubMed central. Specific keywords such as “antibiotic use poultry Bangladesh”, “antibiotic use chicken Bangladesh”, or “antibiotic use livestock Bangladesh”, or “antibiotic use aquaculture Bangladesh”, or “antimicrobial resistance food animals Bangladesh”, or “antibiotic resistance food animals Bangladesh”, or “antimicrobial resistance fish Bangladesh”, or “antimicrobial consumption Bangladesh” were used to search relevant articles. All databases were searched from January to April 2021. We considered those articles, reports, or documents for this review published between January 1972 and March 2021. Based on inclusion criteria, relevant publications were selected for detailed review. The inclusion criteria were included the following characteristics: (a) the study was published in peer-reviewed journals; (b) the study was published during January 1972 to March 2021; and (c) the study reported either antibiotic usage or antimicrobial resistance in food-producing animals. After initial screening, we thoroughly reviewed all selected articles, abstracts, and published documents that were informative and useful for this narrative review. Finally, all authors reviewed the extracted data and revision was made based on the individual’s feedback.

## 3. Results

**Figure 1:**
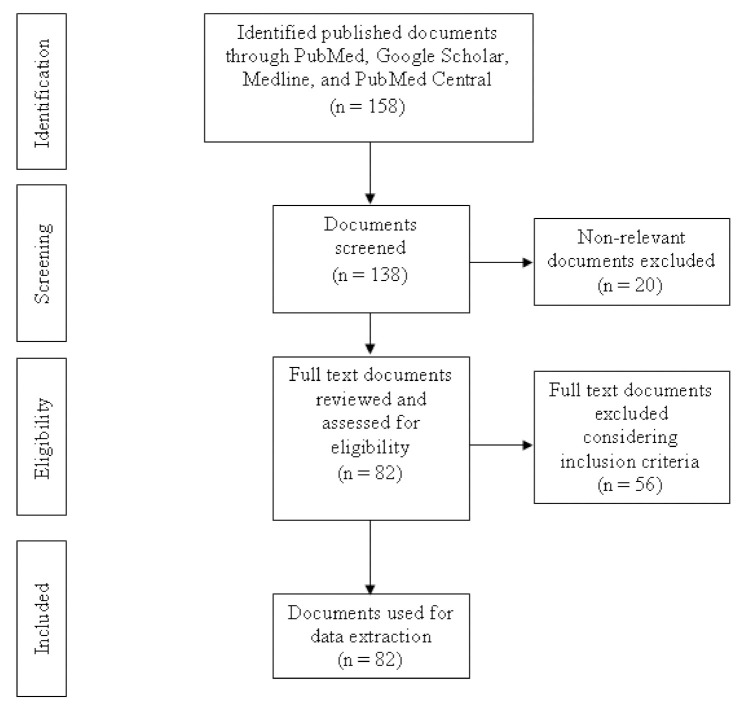
Flow diagram of the document search and selection.

This review describes the antibiotic usage practices and antibiotic resistance in food animal production sectors, challenges, and future directions to combat antibiotic resistance. Initially, we identified more than 158 relevant publications to review only abstract or summary. After initial screening, we selected 82 publications for the full review using inclusion criteria (Figure 1). Antibiotics usage was more commonly reported in commercial chicken and aquaculture than other animal production systems (livestock and backyard poultry). Farmers used antibiotics for both therapeutic and preventive purposes. Many studies detected several antibiotics resistance harmful bacteria in food-producing animals and animal origin foods. 

### 3.1. Antibiotic Usage and Resistance in Commercial Layer Chicken

As per published literature, we found that commercial layer chicken farms use antibiotics for multiple purposes, including therapeutic, growth promotion, and prophylactic purposes. In Bangladesh, the proportion of antibiotic usage during the production cycle of commercial layer farms varied from 54–100%. According to the farmers, 13–43% of the layer farms used antibiotics for prophylactic purposes, and 4% of layer farms used for growth promotion purposes (Table 1) [5,17,27,28]. Ciprofloxacin (37%) was primarily used, followed by amoxicillin (33%), tiamulin (32%), colistin (28%), doxycycline (26%), tylosin (17%), and oxytetracycline (15%) [27]. 

In Bangladesh, many studies reported antibiotic resistance in the commercial layer chicken in Bangladesh (Table 2). Antibiotic-resistant avian pathogenic *Escherichia coli* (APEC) causes significant economic losses in the commercial chicken industry and poses a threat to public health. APEC can transmit from chicken to humans through eggs and meat [32]. The prevalence of *E. coli* reported by a study was 100% in chicken feces and 36% of samples were positive for APEC-associated virulence genes. The APEC-associated virulence genes showed 100% resistance against ampicillin and tetracycline, followed by chloramphenicol (97%), erythromycin (97%), enrofloxacin (56%), norfloxacin (50%), ciprofloxacin (50%), streptomycin (19%), colistin (11%), and gentamicin (8%) [33]. Another study showed a different pattern of resistance where *E. coli* isolates showed resistance to tetracycline (46%), trimethoprim–sulfamethoxazole (27%), nalidixic acid and ampicillin (26%), streptomycin (21%), ciprofloxacin (13%), chloramphenicol (9%), nitrofurantoin and gentamicin (2%) [34]. Poultry and poultry products (meat and eggs) are one of the important sources of human salmonellosis [35]. The prevalence of *Salmonella enterica* in layer chicken was 40%. The isolated strains of *Salmonella* were found resistant against penicillin (100%) and nalidixic acid (100%), followed by sulfamethoxazole-trimethoprim (55%), ampicillin (40%), and amoxicillin (25%) [36]. Colistin is considered one of the last-resort reserved antibiotics for humans. However, colistin is used in animals for therapeutic, prophylactic, and growth promotion purposes [37]. In Bangladesh, a study reported 37.5% of the layer farms used colistin during the chicken production cycle and bacterial isolates detected from fecal samples of layer chicken showed resistance against colistin. The predominant colistin–resistant gene was mcr-1 detected more in commercial chicken than native chicken [30]. 

### 3.2. Antibiotic Usage and Resistance in Commercial Broiler Chicken

Antibiotics are often used in the broiler production systems in Bangladesh. Antibiotics are used to treat clinically sick chickens as well as growth promotion and prophylactic purposes [17,20]. The majority of the broiler farms (95–100%) of the broiler farms administered antibiotics during the production cycle (Table 1) [27]. The most common usage of antibiotics was for therapeutic purposes (44%), followed by prophylactic (32%), and growth promotion (8%) [17]. Many farmers use combination form antibiotics for their broiler chicken, and the majority (80%) of them administered multiple antibiotics [17,20]. Tetracycline, colistin, ciprofloxacin, tylosin, neomycin, amoxicillin, trimethoprim, sulfonamides, doxycycline, erythromycin, and tiamulin were identified as common antibiotics [17,20,27].

In Bangladesh, several epidemiological studies reported antibiotic resistance in commercial broiler chicken (Table 2). *E. coli* was highly prevalent in broiler chicken. A study detected 100% of cloacal swabs collected from broiler chicken tested positive for *E. coli*. *E. coli* isolates from apparently healthy broiler chicken were found 100% resistant to ampicillin and tetracycline followed by sulfamethoxazole–trimethoprim (95%) and nalidixic acid (92%) [39,42]. The commonly detected oxytetracycline resistant genes were tetA, tetB and tetC gene [38]. Another study reported 55% prevalence of Extended-Spectrum Beta-Lactamase (ESBL)-producing *E. coli* in broiler ceca and feces at households, farms, and live poultry markets. The majority (71%) of the ESBL-producing *E. coli* isolates showed resistance against fluoroquinolones and cefepime, followed by sulfonamides (65%) and aminoglycosides (31%) [20]. Avian origin multi-drug resistant *Salmonella* is one of the leading causes of food-borne illness in humans [44]. *Salmonella* was detected in 34% cloacal swabs of broiler chicken, and the majority of the *Salmonella* serovar was *S. enterica* serovar Typhimurium. *Salmonella* isolates showed the highest resistance to tetracycline (97%), followed by chloramphenicol (94%), ampicillin (83%), and streptomycin (77%) [43]. Colistin use in broiler production was not uncommon. There was an evidence of *E. coli* isolates that carried colistin-resistant mcr-1 genes and some of them showed resistance against tetracycline, and Beta-Lactam antibiotics [30,31]. *Campylobacter jejuni* and *Campylobacter coli* were highly prevalent in broiler chicken and many isolates showed resistance against amoxicillin, streptomycin, tetracycline, erythromycin, ciprofloxacin, norfloxacin, and azithromycin [40,45]. 

### 3.3. Antibiotic Usage and Resistance in Aquaculture 

Bacterial infection in fish is common in Bangladesh. More than 80% of the shrimp hatcheries used antibiotics both for treatment and prophylactic purposes. Oxytetracycline, chloro-tetracycline, amoxicillin, erythromycin, ciprofloxacin, co-trimoxazole, sulfadiazine and sulfamethoxazole, chloramphenicol, and prefuran were identified as commonly used antibiotics (Table 3) [46,47,48,49]. 

The occurrence of antibiotic-resistant bacteria in fish and freshwater was not uncommon in Bangladesh (Table 4). A previous study identified 58 bacterial isolates in fish samples belong to nine different genera of bacteria. The most common isolated bacteria were *Klebsiella* spp., *Pseudomonas* spp., *Staphylococcus aureus*, *Vibrio* spp., and *E. coli*. Most of the isolated bacteria were 100% resistant against tetracyclines, penicillins, cephalosporins, aminoglycosides and macrolides; 80% resistance against sulfanilamide and fluoroquinolones [50]. *Pseudomonas fluorescens* cause hemorrhagic septicemia in fish and *P. fluorescens* isolates were detected in infected carp and catfish. The majority of the isolates (80%) were showed resistance against chloramphenicol, sulfamethoxazole, erythromycin, and cephradine [51]. *Listeria* spp. detected in fishes were found resistant against penicillin G and ampicillin, whereas *Staphylococcus aureus* showed resistance against trimethoprim, erythromycin, sulfamethoxazole, and *E. coli* showed resistance against erythromycin and polymyxin B [52]. *Vibrio parahaemolyticus* was detected in 60% of samples from the aquaculture system, and most of the *V. parahaemolyticus* isolates were found resistant to ampicillin and amoxicillin (94%), followed by cefotaxime (29%), and ceftriaxone (18%) [53].

### 3.4. Antibiotic Usage and Resistance in Duck, Pigeon, Quail, and Sonali Chicken

In Bangladesh, we did not find antibiotic usage data for duck, pigeon, and quail. However, many studies reported antibiotic-resistant bacteria in duck, pigeon, and quail. Salmonella prevalence was 40% in duck, and most of the *Salmonella* isolates showed resistance against amoxicillin, tetracycline, and tobramycin [54]. The prevalence of *Pasteurella multocida* infection in ducks was 34% and 100% of isolates showed resistance against penicillin G [55]. Antibiotics resistance was reported in quail. *E. coli* isolates from quail showed resistance against amoxicillin, gentamycin, nalidixic acid, and tetracycline, whereas *Salmonella* spp. showed resistance against amoxicillin, ampicillin, erythromycin, gentamycin, kanamycin, nalidixic acid, and tetracycline. *Pasteurella* spp. were found resistant against erythromycin, sulfaamethoxazole, and tetracycline [56]. The isolated strains of *Salmonella* in pigeons showed resistance against ampicillin, cephalexin, tetracycline, erythromycin, and colistin sulfate [57]. Thus far, no studies were conducted to determine antibiotic usage and resistance in Sonali chicken.

### 3.5. Antibiotic Usage and Resistance in Livestock 

In livestock, antibiotics are mainly used to treat diseases. Two hospital-based studies reported that antimicrobials were prescribed to treat 56–66% of sick animals [58,59]. The most commonly prescribed antibiotics were streptomycin–penicillin (31%) followed by sulfadimidine (14%), amoxicillin (11%), gentamicin–sulfadiazine–trimethoprim combination (9%), and tylosin (1%) [58]. Shiga toxin-producing *E. coli* (STEC) and enterotoxigenic *E. coli* (ETEC) showed multidrug-resistant against erythromycin, trimethoprim–sulfamethoxazole, azithromycin, cephalothin, ciprofloxacin, and nalidixic acid [60]. *Salmonella* strains isolated from diarrheic cattle showed resistance to azithromycin, tetracycline, and erythromycin [61]. *Staphylococcus aureus* isolated from dairy cows showed resistance to oxytetracycline [62]. *Staphylococcus* spp., and *Bacillus* spp. showed a high level of resistance to ampicillin, amoxicillin, and streptomycin in goats [63]. *Listeria monocytogenes* isolates from cattle showed 100% resistance against penicillin, imipenem, and amoxicillin [64]. 

### 3.6. Antibiotic Residues and Resistance in Animal-Origin Foods

In Bangladesh, many studies reported antibiotic residues and antibiotic-resistant bacteria in animal-origin foods. Several antibiotics, including amoxicillin, oxytetracycline, ciprofloxacin, enrofloxacin, fluoroquinolones, sulfonamides, and polymyxin residues were detected in raw meat and liver samples of chicken [17,65]. Amoxicillin residue and oxytetracycline residues were identified in freshwater fish samples [65]. Another study detected oxytetracycline and ciprofloxacin residues in 18% of milk collected from commercial dairy farms [66].

Frozen chicken meat samples were tested positive for ESBL producing *E. coli* and Methicillin-resistant *Staphylococcus aureus* (MRSA) [67,68,69,70]. The *E. coli* isolated from chicken meat samples were found resistant against multiples antibiotics, such as oxytetracycline, amoxicillin, ampicillin, trimethoprim–sulfamethoxazole, pefloxacin, tetracycline, and carbapenems [69,70]. *S. aureus* showed wide-ranging resistance against cefoxitin, nalidixic acid, ampicillin, oxacillin, colistin, amoxicillin–clavulanic acid, amoxicillin, penicillin-G, cloxacillin, oxytetracycline, and cefixime [71]. Studies detected *Salmonella* and *S. aureus* in chicken egg content and eggshell surface. The *Salmonella* isolates showed significant resistance against amoxicillin and ampicillin, whereas *S. aureus* isolates were found resistant to amoxicillin, nalidixic acid, and penicillin [68,72]. Another study detected *Staphylococcus* spp., *Alcaligenes* spp., *Klebsiella* spp., and *Pseudomonas* spp. in animal origin frozen foods collected from supermarkets. Contamination was mostly found in chicken nuggets, and the isolated bacteria showed resistance against cefixime, chloramphenicol, nalidixic acid, and azithromycin [73]. A study detected 35% of milk samples collected from cows and buffaloes were tested positive for *S. aureus,* and 9% were positive for *E. coli*. Most of the *S. aureus* and *E. coli* isolates were resistant to gatifloxacin [74]. 

## 4. Discussion

This report describes the antibiotic usage practices and antibiotic resistance in food-producing animals in Bangladesh. Many studies raised significant concerns about the occurrence of antibiotic residues in chicken meat and antibiotic resistance in the commercial chicken production system. Most commercial chicken farms had a history of extensive antibiotics usage for treatment, prophylactic, and growth promotion purposes. Similar practices of antibiotic use in China were reported in commercial chicken farms where amoxicillin, norfloxacin, ofloxacin, ceftriaxone, and oxytetracycline were commonly used antibiotics [75]. The use of antibiotics as prophylactic was reported in other countries including Thailand, Vietnam, and China [3,4,75]. The World Organization for Animal Health (OIE) and WHO have suggested avoiding antibiotics usage in healthy food-producing animals to prevent the emergence and spread of antibiotic resistance [7,76].

Irrational and excessive usage of antibiotics in humans and food-producing animals increases the risk of antibiotic resistance worldwide [77]. In Bangladesh, many commercial chicken farmers used antibiotics for growth promotions purposes. Though very limited information was generated from Bangladesh on antibiotics use in freshwater aquaculture, the high prevalence of multidrug-resistant bacteria and antibiotic residues in fish samples indicate frequent and indiscriminate use of antibiotics in aquaculture. TheFood and Agriculture Organization (FAO), OIE, and WHO have identified antimicrobial residues and resistance as significant hazards for aquaculture [78]. The regular use of antibiotics as a growth promoter and prophylactic in aquaculture sectors in Bangladesh may contribute to the development of antibiotic resistance bacteria. Commercial chicken and fish farmers should be more cautious regarding antibiotic use considering the impact of unnecessary usage of antibiotics, and should not use antibiotics routinely to promote growth and prevent diseases in healthy chickens and fish.

The knowledge level of small-scale poultry farmers in low-middle-income countries on antimicrobial use and the emergence of antimicrobial resistance is insufficient [79]. In Bangladesh, drivers of antibiotic resistance in the commercial chicken and aquaculture production system may be associated with excessive use of antibiotics, and use of antibiotics as prophylactic and growth promoters. The occurrence of the colistin-resistant mcr genes in layer chicken may lead to the development of resistance in humans. Many types of antibiotics are used to treat and prevent bacterial infections. The aquatic environment can be affected by excessive antibiotic use that may play a vital role in harboring and disseminating antibiotic resistance across various ecosystems [80]. The presence of antibiotic residues in fish raises concerns about developing resistance. The role of antibiotic residues in meat, milk, and egg can alter microflora and develop resistance against antibiotics [81]. The frequent use of critically important antibiotics, including quinolones, the third or fourth generation of cephalosporins, and polymyxins in animal production sectors needs to be controlled to reduce the risk of antibiotic resistance in public health. 

In Bangladesh, indiscriminate use of antibiotics by unqualified providers, irrational antibiotic dispense by animal feed dealers, aggressive marketing, over-the-counter dispensing by drug sellers, and absence of antibiotic use guidelines, and poor implementation of regulations may contribute to developing antibiotic resistance. Veterinary practitioners primarily treat animals based on presumptive diagnosis because of inadequate diagnostic facilities. A hospital-based study identified more than 90% of sick animals were treated with antibiotics by the veterinarian [82]. Adequate laboratory diagnostic facilities can help veterinarians and animal farmers to diagnose actual causes that may reduce unnecessary antibiotic use. Awareness of the rational use of antibiotics in food animals, safe food handling and safe cooking practices is crucial to reduce the risk from pathogenic antibiotic resistance bacteria originating from animal origin foods.

## 5. Conclusions and Recommendations

Antibiotics usage in commercial chicken and aquaculture production sectors was extensive in Bangladesh. Non-therapeutic usage of antibiotics in commercial chicken and fish has raised significant concerns about the development of antibiotic resistance. Since antibiotic resistance is a multi-faceted problem, Bangladesh needs well-coordinated efforts through the One Health approach to combat antibiotic resistance. Government should develop and implement strict guidelines urgently for the use of antimicrobial agents in food animals. Comprehensive antibiotic administration monitoring systems can be helpful to minimize the emergence of antibiotic resistance. An extensive awareness program for farmers is crucial to reduce the unnecessary use of antibiotics in healthy chickens. Intensive awareness training program for farmers, feed dealers and drug sellers may be helpful to raise awareness on good farm practices, standard biosecurity practices and their benefit, personal hygiene and the prudent use of antibiotics. Adequate laboratory diagnostic facilities need to be established at the central to the root level to help veterinarians and animal farmers for the prudent use of antibiotics. Finally, more research is required to generate more specific data on antibiotic usage in animal sectors and detect the emergence of antibiotic resistance in animals and humans.

## Figures and Tables

**Table 1:** Summary of studies included for exploring antibiotic use in commercial layer and broiler chicken production system, Bangladesh.

Study Author, Year	Study Description	Key Findings
Tasneem Imam et al., 2020 [27]	A cross-sectional study to collect information on antimicrobial usage in commercial broiler and layer farms.	One hundred percent of broiler and layer farms used antibiotics in the current production cycle. Moreover, 13% of the layer farmers used antibiotics for prophylactic purposes. Ciprofloxacin (37%) was mostly used, followed by amoxicillin (33%), tiamulin (32%), colistin (28%), doxycycline (26%), tylosin (17%), and oxytetracycline (15%); 18% of the broiler farmers used antibiotics for prophylactic purposes. Colistin, ciprofloxacin, tylosin, neomycin, amoxicillin, trimethoprim, sulfonamides, doxycycline, and tiamulin were used more frequently.
K. B. M. Saiful Islam et al., 2016 [17]	Surveyed 73 poultry farms from different regions of Bangladesh to explore the pattern of antibiotic usage.	One hundred percent of farms used at least one antibiotic in the last 12 months.
Emily K.Rousham et al., 2021 [20]	A cross-sectional survey to detect ESBL-EC carriage in adults from three communities with close human–poultry interactions; backyard poultry in rural households; small commercial broiler poultry farms; and urban food markets that sell live poultry with on-site slaughtering and processing.	Ninety-five percent of broiler farms used antibiotics, and 80% of them administered multiple antibiotics. Tetracycline (63%) mainly was administered, followed by ciprofloxacin (55%) and enrofloxacin (55%), erythromycin (38%), tylosin (38%), and colistin sulfate (15%).
Jannatul Ferdous et al., 2019 [28]	Surveyed 120 small–scale layer farms to collect data on antibiotic usage.	All farms administered antibiotics in the chicken production cycle. The most common antibiotics were ciprofloxacin (23%), followed by enrofloxacin (18%), amoxicillin (17%), oxytetracycline (11%), sulfa drugs (3%), and norfloxacin (2%); 73% of antibiotics are critically important for humans.
S.T. Tasmim et al., 2021 [5]	A qualitative survey of 70 farmers was conducted to assess poultry farmer’s knowledge and practices regarding antibiotics, antibiotic use, and antimicrobial resistance and to identify the factors.	Fifty-four percent of commercial poultry farmers used antibiotics from the first day of a chicken production cycle, 43% of farmers used antibiotics for preventive purposes, and 4% used antibiotics as a growth promoter.
Amira A. Roess et al., 2013 [29]	Semi-structured in-depth interviews with key informants (female household members, village doctors, pharmaceutical representatives, veterinarians, and government officials) and performed observations at animal health clinics.	Most of the farmers used antimicrobial drugs to treat their backyard animals.
Salequl Islam et al., 2020 [30]	A cross-sectional study to examine the colistin resistance mcr-1 to mcr-5 genes prevalence in bacterial isolates from chicken droppings.	Sixty-seven percent of the broiler farms and 38% of the layer farms used colistin in the chicken production cycle.
Shahana Ahmed et al., 2020 [31]	A study to examine the colistin resistance mcr-1 to mcr-5 genes prevalence in bacterial isolates from broiler chicken.	Sixty-five percent of broiler farms administered colistin in the chicken production cycle.

**Table 2:** Evidence of AMR in commercial layer and broiler chicken production system, Bangladesh.

Study Author, Year	Study Description	Key Findings
Badrul Hasan et al., 2011 [34]	A total of 279 dead or sick broiler and layer chickens of different ages were tested for pathogenic *E. coli* strains and to determine the phenotypic expression of antimicrobial resistance against antibiotics.	More than 55% of *E. coli* isolates were resistant to at least one antibiotic, and 36.6% of the isolates showed resistance against multiple antibiotics.
Samina Ievy et al., 2020 [33]	A total of 99 samples from commercial layer farms were tested to determine the prevalence of avian pathogenic *E. coli* (APEC), the associated virulence genes and their antibiotic resistance profiles.	APEC-associated virulence genes showed 100% resistance against ampicillin and tetracycline, followed by chloramphenicol (97%), erythromycin (97%), enrofloxacin (56%), norfloxacin (50%), ciprofloxacin (50%), streptomycin (19%), colistin (11%), and gentamicin (8%).
Avijit Das et al., 2020 [38]	*E. coli* strains isolated from 30 broiler farms were examined to detect resistance against oxytetracycline and ciprofloxacin.	*E. coli* showed resistance against oxytetracycline (100%) and ciprofloxacin (78%).
Badrul Hasan et al., 2011 [34]	A total of 279 dead or sick broiler and layer chicken of different ages were tested for pathogenic *E. coli* strains, and to determine the phenotypic expression of antimicrobial resistance against antibiotics.	More than 55% of *E. coli* isolates were resistant to at least one antibiotic, and 36.6% of the isolates showed resistance against multiple antibiotics.
Emily K.Rousham et al., 2021 [20]	A cross-sectional survey to detect ESBL-EC carriage in adults from three communities with close human–poultry interactions; backyard poultry in rural households; small commercial broiler poultry farms; and urban food markets that sell live poultry with on-site slaughtering and processing.	A total of 71% of the ESBL-producing *E. coli* isolates showed resistance against fluoroquinolones and cefepime, followed by sulfonamides (65%) and aminoglycosides (31%).
Md. Samun Sarker et al., 2019 [39]	A study to detect *E. coli in* apparently healthy broiler chicken and detect antibiotic-resistant genes.	*E. coli* was detected in 62% of broiler chicken. *E. coli* isolates were 100% resistant to ampicillin and tetracycline followed by sulfamethoxazole-trimethoprim (95%) and nalidixic acid (92%).
Badrul Alam et al., 2020 [40]	A total of 128 cloacal swabs from broiler chicken, 64 poultry feed, 64 drinking water, 64 attendants’ hand rinsed water, and 32 whole broiler carcasses were tested to detect *Campylobacter* spp. All *Campylobacter* spp. strains were tested against eight antimicrobial agents.	Twenty-six percent of samples tested positive for *Campylobacter* spp. A total of 93 isolates of Campylobacter were detected. Among them, 22 isolates of *Campylobacter jejuni* showed resistance against amoxicillin, streptomycin, tetracycline, ciprofloxacin, amoxicillin, norfloxacin, and azithromycin.
Sucharit Basu Neogi et al., 2020 [41]	A total of 224 samples from 7 hatcheries, 9 broiler farms and 4 live bird markets were tested to examine the occurrence and MDR patterns of *Campylobacter* spp.	Thirty-two percent of samples were tested positive for *Campylobacter* spp.; 49% of strains of *C. jejuni* showed resistance to three or more antimicrobials, including tetracycline, amoxicillin, streptomycin, fluoroquinolones, and macrolides.
Muha. Ajijur Rahman Al Azad et al., 2019 [42]	A study to detect E. coli strains from live broiler chickens and to determine their susceptibility and resistant patterns to selected antimicrobial agents.	Detected 100% prevalence of *E. coli* in cloacal swabs. All *E. coli* isolates were found resistant against ampicillin, tetracycline, streptomycin, ciprofloxacin, erythromycin, and trimethoprim–sulfamethoxazole. Colistin sulfate and gentamicin showed the highest susceptibility to antibiotics.
Shanzida Binte Alam et al., 2019 [43]	A total of 100 samples from broiler chickens were tested to detect multi drug resistant *Salmonella* along with the resistance pattern.	*Salmonella* isolates showed the highest resistance to tetracycline (97%), followed by chloramphenicol (94%), ampicillin (83%), and streptomycin (77%).
Salequl Islam et al., 2020 [30]	A cross-sectional study to examine the colistin resistance mcr-1 to mcr-5 genes prevalence in bacterial isolates from chicken droppings.	A total of 64 isolates from commercial chicken (broiler and layer) and 28 isolates from native chicken showed colistin-resistance.
Shahana Ahmed et al., 2020 [31]	A study to examine the colistin resistance mcr-1 to mcr-5 genes prevalence in bacterial isolates from broiler chicken.	Twenty-five percent of *E. coli* isolates carried colistin-resistant mcr-1 genes. *E. coli* isolates also showed resistance against Tetracycline, and Beta-Lactam antibiotics.

**Table 3:** Summary of studies included for exploring antibiotic use in aquaculture, Bangladesh.

Study Author, Year	Study Description	Key Findings
Sheikh Aftab Uddin et al., 2006 [49]	A cross-sectional study to know the pattern of antibiotics and other chemical use in shrimp hatcheries.	Eighty percent of shrimp hatcheries used antibiotics. Chloramphenicol, erythromycin, oxytetracycline, and prefuran were commonly used antibiotics.
Md. Abu Kawsar et al., 2019 [46]	A study to assess the aqua drugs and antibiotics used in aquaculture.	Fifty-two percent of farmers used erythromycin, 18% used ciprofloxacin and 10% used oxytetracycline.
A.K. Jilani Chowdhury et al., 2012 [48]	A cross-sectional study to find out different chemicals used in fish hatcheries, nurseries, and culture farms.	Fourty-seven percent of fish farmers used oxytetracycline to treat diseases.

**Table 4:** Evidence of AMR in aquaculture, Bangladesh.

Study Author, Year	Study Description	Key Findings
Farzana Ehetasum Hossain et al., 2018 [50]	A study to investigate the presence of bacteria in fish samples and to analyze multidrug resistance pattern of the bacteria.	Most of the bacterial isolates in drug used ponds were 100% resistant against tetracyclines, penicillins, cephalosporins, aminoglycosides, and macrolides; 80% resistance against sulfanilamide and fluoroquinolones.
Foysal MJ et al., 2011 [51]	A study to isolate and identify *Pseudomonas fluorescens* from bacterial hemorrhagic septicemia infected carp and catfish to find out their antibiotic sensitivity pattern.	Most of the *Pseudomonas fluorescens* isolates (80%) were found resistant to chloramphenicol, followed by sulfamethoxazole (70%), erythromycin (60%), and cephradine (30%).
Tasnia Ahmed et al., 2013 [52]	Tested export quality shrimp samples to detect *Listeria* spp., *Staphylococcus aureus*, and *E. coli* and to determine the antibiotic resistance.	*Listeria* spp. was resistant against penicillin G and ampicillin, whereas *S. aureus* showed resistance against trimethoprim, erythromycin, sulfamethoxazole, and *E. coli* showed resistance against erythromycin and polymyxin B.
Abu Baker Siddique et al., 2021 [53]	A total of 216 samples from water, sediment, *Oreochromis niloticus* (tilapia fish), *Labeo rohita* (rui fish), and *Penaeus monodon* (shrimp) were tested to detect *Vibrio parahaemolyticus* and to analyze multidrug resistance pattern of the bacteria.	Sixty percent of samples from the aquaculture system tested positive for *V. parahaemolyticus.* Most of the *V. parahaemolyticus* isolates were found resistant to ampicillin and amoxicillin (94%), followed by cefotaxime (29%), and ceftriaxone (18%).

## Data Availability

All data are available from the corresponding author after reasonable request.
